# The Taxonomic Position and Phylogenetic Relationship between *Digramma interrupta* and *Ligula intestinalis* Based on Morphological and Molecular Diagnosis

**Published:** 2018

**Authors:** Emad AHMADIARA, Seyed Hossein HOSSEINI, Fatemeh JALOUSIAN, Hossein Ali EBRAHIMZADEH MOUSAVI

**Affiliations:** 1. Dept. of Parasitology, School of Veterinary Medicine, University of Tehran, Tehran, Iran; 2. Dept. of Aquatic Animal Health, School of Veterinary Medicine, University of Tehran, Tehran, Iran

**Keywords:** *Ligula intestinalis*, *Digramma interrupta*, Morphology, Molecular diagnosis, Iran

## Abstract

**Background:**

The position of *Digramma interrupta* remains disputable as it was raised by Cholodkovsky from *Ligula alternans*. This study aimed to survey the evolutionary relationships and the taxonomic position of *D. interrupta* and *L. intestinalis*. It also intended to support or reject the validity of *D. interrupt* as an independent genus and its correlation with *L. intestinalis* on the basis of their morphological characteristics and a study on molecular data.

**Methods:**

Overall, 1301 fish varieties, including 883 *Alburnoides bipunctatus* and 418 *Abramis brama*, were collected from north and north-western parts of Iran. *A. bipunctatus* samples were obtained from fresh water sources of the Maragheh dam (northwest) and the Ramesar Lake (north). Moreover, samples of *A. brama* were captured from the Aras Dam (northwest) and the Bandar-e-Anzali lagoon (north). PCR was used to generate a fragment spanning two independent ITS-inclusive parts: ITS1-5.8S and ITS2 with two pairs of primers.

**Results:**

Nucleotide variation between *L. intestinalis* and *D. interrupta* samples amounts to about 3% to 7%. Between samples of *L. intestinalis* and GenBank data, and also between *D. interrupta* specimens and GenBank data, the diversity was seen for about 1% to 3%. Moreover, about 1% to 4% nucleotide variation was seen only in *L. intestinalis* samples caught from the same host, which could be supplementary to the presence of a species and/or strains in this genus.

**Conclusion:**

Maybe *D. interrupta* was just a rare diplogonadic form of the *Ligula* species, not a different genus and not synonymous with the *Ligula* genus, but only another species of the *Ligula* genus.

## Introduction

Members of diphyllobothridae are the most significant cestoda that infect fish as the intermediated host in the plerocercoid phase (1). This family has significant genera like *Ligula intestinalis*, and *Digramma interrupta* is distributed all around the world.

The position of *D. interrupta* in diphyllobothridae family remains disputable since it was raised by Cholodkovsky from *L. alternans*. Morphological differences, with one set of reproductive organs in *L. intestinalis* and two sets of this in *D. interrupta,* present the most important differences between them. However, the morphological features are not completely reliable to distinguish genus from each other (2).

One of the aims of this study was support or reject the validity of *D. interrupta* and its correlation with *L. intestinalis* on the basis of morphological characteristics and a study of molecular data based on entire internal transcribed spacer of the ribosomal DNA (ITS rDNA) that contains ITS1- 5.8S and ITS2. In addition, this study intends to assess the genetic diversity of *L. intestinalis* or *D. interrupta* from different hosts and geographical regions.

## Materials and Methods

### Samples collection

**Fig. 1:**
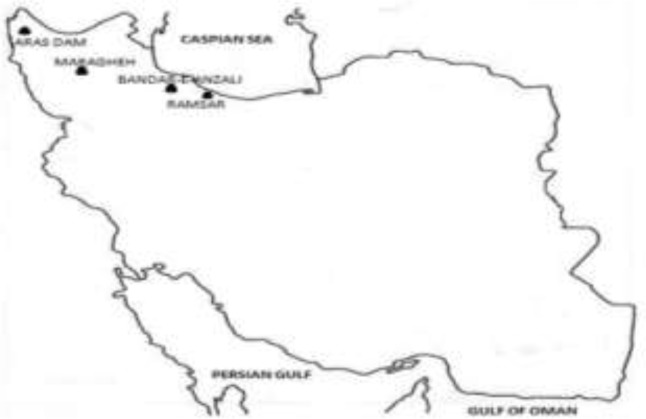
Localities in Iran where specimens of *Ligula intestinalis* and *Digramma interrupta* were collected

Overall, 1301 fish varieties, including 883 *Alburnoides bipunctatus (A. bipunctatus)* and 418 *Abramis brama (A. brama),* were collected from north and north-western parts of Iran*. A. bipunctatus* samples were obtained from fresh water sources of the Maragheh dam (northwest) and the Ramesar Lake (north). Moreover, samples of *A. brama* were captured from the Aras Dam (northwest) and the Bandar-e-Anzali lagoon (north) (Fig. 1). The samples were put into ice boxes and immediately transferred to the laboratory; they were isolated from body cavities of infectious fish and preserved either by being placed in 96% ethanol or being stored at −20 *°*C for morphological and molecular analysis.

### Morphological characterizations

The samples’ terminal segments were stained with aceto-carmine and mounted with the Canada balsam. The specimens were identified as *L. intestinalis* and *D. interrupta* by using characters that are suitable for species identification according to taxonomic keys (3).

**Fig. 2:**
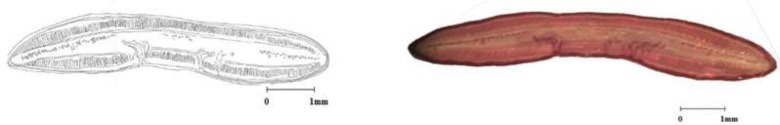
Cross section of *Digramma interrupta* with two sets of reproductive organs

**Fig. 3:**
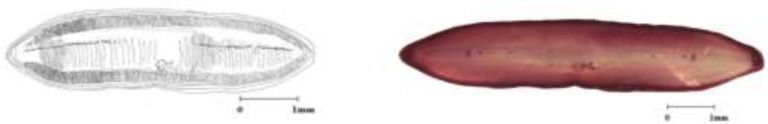
Cross section of *Ligula intestinalis* with one set of reproductive organ

**Fig. 4:**
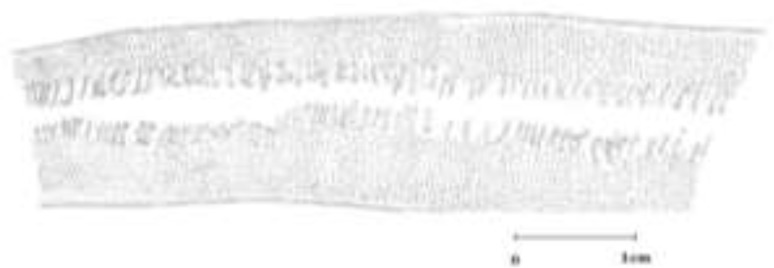
Longitudinal sections of *Digramma interrupta* with two rows of reproductive organ

**Fig. 5:**
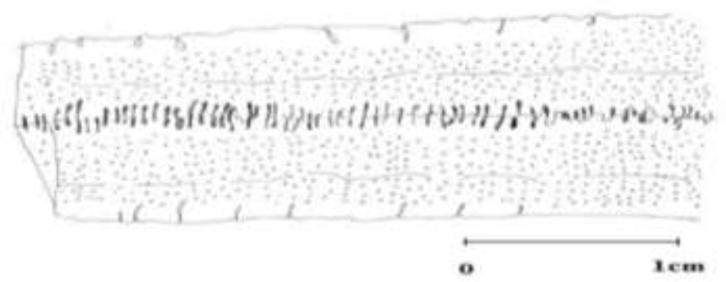
Longitudinal sections of *Ligula intestinalis* with one row of reproductive organ

The morphological characterization of plerocercoid was completed by observing them under a light microscope equipped with the camera lucida. Then, for having an accurate survey, we draw the schematic characteristics of the specimens, and transferred them onto talk paper and scanned for more accurate analysis (Figs. 2–5).

### DNA extraction

Deoxyribonucleic acid (DNA) was extracted by using a DNA isolation kit (Qiagen, Germany) according to the manufacturer’s instructions.

### PCR amplification

PCR was used to generate a fragment spanning two ITS-inclusive independent parts, namely ITS1-5.8S and ITS2 with two pairs of specific primers. These primers were designed by Vector NTI11. The first for the 891bp fragment comprised ITS1-5.8S, and second for the 421bp sequence-long inclusive ITS2 locus (Table 1). The PCR product was purified by using the PCR purification kit (Qiagen, Germany). The sequencing was performed from both sites of each PCR products by Kawsar Biotech Company in Iran on the basis of the Sanger method (1977). The sequences were analyzed by using the Chromas version 1.3 software and CLC Main Workbench 5, and they were compared with samples registered in GenBank by using the ‘Basic Local Alignment Search Tool’ (BLAST) program. The phylogenetic tree was designed by MEGA version 5.0 software.

**Table 1:** Primer 1 (DigIr F 5′ & DigIr R 5′) for 891bp fragment consist of ITS1, 5.8S and ITS2; Primer 2 (Ligir2F 5′ & Ligir2R 5′) for 421bp sequence long include ITS2 locus

***Primer 1, product size: 890bp***
DigIr F 5′ - CACGTTCCGTCTA TATGCGC-3′
DigIr R 5′- GGCAGCATCTCGCTTAAATG-3′
**Primer 2, product size:421bp**
Ligir2F 5′ - TGGCGGGAAAACTCGGGCTT-3′
Ligir2R 5′ - GCCGCCAACCACCAACAG-3′

## Results

All isolates morphologically derived from *A. bipunctatus* were distinguished from *L. intestinalis,* and all specimens of *A. brama* were determined to be *D. interrupta*. From 883 specimens of *A. bipunctatus* and 418 collected samples of *A. brama*, 558 (63.19%) and 67 fishes (16%) were infected, respectively (Table 2).

**Table 2:** Sampling localities and coordinates of *Alburnoides bipunctatus* and *Abramis brama* in this study

***Sampled fish***	***Province***	***Locality***	***Geographical position***	***Rate of infection***	***Plerocercoid detected***
*A. bipunctatus*	East Azerbaijan	Maragheh Dam	Northwest	63.19%	*L. intestinalis*
*A. brama*	West Azerbaijan	Aras Dam	Northwest	16%	*D. interrupta*
*A. bipunctatus*	Guilan	Bandare-E-Anzali lagoon	North	-	-
*A. brama*	Mazandaran	Ramesar lake	North	-	-

After blast, the data, according to the ITS locus, 13 isolates were determined from *D. interrupta* and 10 samples were determined *L. intestinalis.* All the molecular results were coordinated with the morphological outcome.

All 13 *D. interrupta* samples were registered in the GenBank under accession numbers KC900982-KC900994. Ten samples were referred to as *L. intestinalis* registered in the Gen-Bank under accession numbers KC900972-KC900981. Nucleotide variation between the *L. intestinalis* and *D. interrupta* samples was about 3% to 7%.

Between *L. intestinalis* samples in this survey and the GenBank samples, and also between *D. interrupta* samples and the GenBank samples, nucleotide variation was about 1% to 3%. Finally, *L. intestinalis* samples were caught in Iran from the same host and nucleotide variations of about 1% to 4% were seen among them wonderfully.

**Fig. 6:**
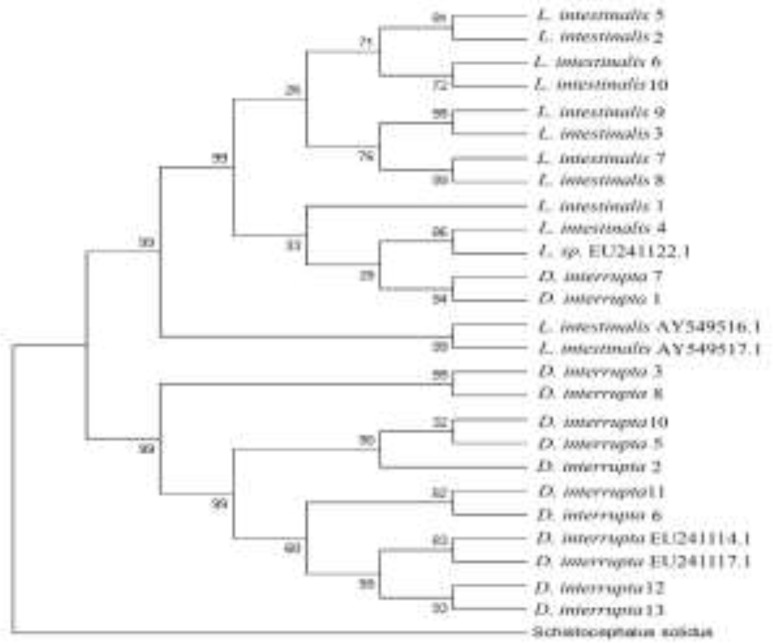
Maximum likelihood phylogenetic tree of *Ligula intestinalis* and *Digramma interrupta* based on ITS2 sequences. Phylogenetic trees were obtained by comparing the ITS2 query sequences of *L. intestinalis* and *D. interrupta* with those of other Cestoda species available in GenBank based on maximum parsimony. Similar topology was observed among the trees obtained by distance-based (NJ) tree building methods in phylogenetic analysis using MEGA7 software. The species included in the maximum parsimony analysis mainly clustered into two major clades and *Schistocephalus solidus* (Pseudophyllidea: Diphyllobothriidae) rooted as an out-group and indicates its evolutionary relationships

The results of the genealogy tree based on the ITS2 locus display that two clades were obvious. One containing *L. intestinalis* and *D. interrupta* samples from present study with five isolates of this other *L. intestinalis* and *D. interrupta* that registered in GenBank, while another clade contains genus *Schistocephalus solidus* that is available in GenBank. All samples caught in this study, including 10 isolates of *L. intestinalis* and 11 isolates of *D. interrupta,* along with five isolates from GenBank were located in the same clade and so we could call them monophyletic. *S. solidus* is located in a different clade (Table 3, Fig. 6).

**Table 3:** Phylogenetic data collection to assess relationships between *Ligula intestinalis* and *Digramma interrupta*

***No.***	***Sample name***	***Locality***	***Host***	***Accession no.***
1	*L. intestinalis* 1	Iran	*A. bipunctatus*	KC900972.1
2	*L. intestinalis* 2	Iran	*A. bipunctatus*	KC900973.1
3	*L. intestinalis* 3	Iran	*A. bipunctatus*	KC900974.1
4	*L. intestinalis* 4	Iran	*A. bipunctatus*	KC900975.1
5	*L. intestinalis* 5	Iran	*A. bipunctatus*	KC900976.1
6	*L. intestinalis* 6	Iran	*A. bipunctatus*	KC900977.1
7	*L. intestinalis* 7	Iran	*A. bipunctatus*	KC900978.1
8	*L. intestinalis* 8	Iran	*A. bipunctatus*	KC900979.1
9	*L. intestinalis* 9	Iran	*A. bipunctatus*	KC900980.1
10	*L. intestinalis* 10	Iran	*A. bipunctatus*	KC900981.1
11	*D. interrupta* 1	Iran	*A. brama*	KC900982.1
12	*D. interrupta* 2	Iran	*A. brama*	KC900983.1
13	*D. interrupta* 3	Iran	*A. brama*	KC900984.1
14	*D. interrupta5*	Iran	*A. brama*	KC900986.1
15	*D. interrupta* 6	Iran	*A. brama*	KC900987.1
16	*D. interrupta* 7	Iran	*A. brama*	KC900988.1
17	*D. interrupta* 8	Iran	*A. brama*	KC900989.1
18	*D. interrupta* 10	Iran	*A. brama*	KC900991.1
19	*D. interrupta* 11	Iran	*A. brama*	KC900992.1
20	*D. interrupta* 12	Iran	*A. brama*	KC900993.1
21	*D. interrupta* 13	Iran	*A. brama*	KC900994.1
22	*S. solidus*	Norway	*Gasterosteus aculeatus*	AY549508.1
23	*L. intestinalis*	Turkey	*Silurus glanis*	AY549516.1
24	*L. intestinalis*	Turkey	*Chalcaburnus* sp	AY549517.1
25	*L. colymbi*	Poland	*Gavia stellata*	EU241090.1
26	*D. interrupta*	Russia	*Hemiculter lucidus*	EU241114.1
27	*D. interrupta*	Russia	Hemiculter lucidus	EU241117.1

## Discussion

Studies on the distribution of *D. interrupta* and *L. intestinalis* in Iran showed that most such reports have been from the northwest and western parts, while only a few reports came from the northern area (4, 5). In this study, none of the collected samples from the northern part was infected, but, in contrast, the infection rate was remarkably up in the north-western part of country. Host specificity and isolated lineages by geography were the results of adjustment to local host fauna (6). Moreover, *L. intestinalis* is remarkably host-specific remarkably in Kenya (7). According to the result of this study, infection with plerocercoid was highly correlated with habitat in northwest Iran.

Climatic conditions in the north-western part of the country are more conducive to these parasites, while another probable reason could be the neighborhood of Turkey. Since Turkey is a good source of pollution by Ligulidae plerocercoid, maybe water imported from Turkey can be attributed to infection of water by plerocercoid in northwest Iran (8). Another reason could be migratory birds that could transmit the infection in nearby locations in two adjacent countries.

Host specificity probably exists in Iran because *D. interrupta* was detected only from *A. brama*, in Russia (9). In addition, similar to a survey (5), results from northwest Iran showed detection of plerocercoid of *L. intestinalis* from *A. bipunctatus*. However, the morphological difference could be used to detect the genus and species from each other, but it is not enough and an accurate meter is needed for distinguishing the species and lineage from each other. Therefore, over the last two decades, molecular studies have helped scientists to improve and revise their information about the taxonomical status of parasites and their phylogeny.

Despite various studies on the epidemiological aspect of this Cestoda, any molecular characterization is not found in Iran and also in the entire Middle East zone. Thus, there is lack of sufficient information about the molecular characteristics of this Pseudophyllidean Cestoda in this zone. It confirms the necessity of a study on molecular and morphological features of this genus, and another genus of this family, as well as their true taxonomical station in this region.

Liao and Liang (10) watched in *Digramma* the transitional reproductive organ structure. A single set of reproductive organs was located at the anterior end of the larva in the carassian intermediate hosts and also two rows of this were located at the posterior end. Sexual dimorphism was reported in *D. interrupta* in *A. brama* the reproductive organs are found in one row, whereas two rows occur in those from carp (9). Maybe *D. interrupta* was just a rare diplogonadic form of the *Ligula* species (2). In *L. intestinalis* dimorphism with some of them debating on the potential existence of this species in the *Ligula* genus. For example, based on ITS region and 28S rRNA, verified that plerocercoid of *L. intestinalis* specimens from *Rutilus* and *Gobio* may represent different strains or species (11). Despite recognition of species based on morphological features appropriate for the discrimination between two genera (3), the morphological specification of them is not reliable and raises much confusion concerning *D. interrupta* validity because of the transitional shape of reproductive organs in the proglottids of these two genera.

In the present study, *D. interrupta* exhibited identical sequences with *L. intestinalis* with about 1% heterology based on the ITS1-5.8S regions analysis, whereas a high degree of genetic diversity of about 3% to 7% has seen based on the ITS2 locus. The present study also suggests that ITS2, unlike ITS1-5.8S sequences, can act as a useful genetic marker.

There is not any yardstick for identifying species or genus borders by using the result of the DNA-sequence distinction, with a value of divergence based on ITS2 sequences, *D. interrupta* is almost different from *L. intestinalis*. Therefore, with nucleotide variation levels between *L. intestinalis* and *D. interrupta* specimens, besides morphological differences, it cannot be verified whether *D. interrupta* is a synonym of the genus *L. intestinalis,* against the theory of some researchers (10, 11).

On the other hand, they are different genera because of low levels of nucleotide variation and unstable morphological difference between them. Maybe polymorphism seen in *L. intestinalis* and *D. interrupta* is present only in some global locations (10, 12). Besides low levels of nucleotide variation (else two samples that have 6% and 7% diversity, the other differences would amount to (3% to 4%), we reach and verify the theory that maybe *D. interrupta* was just a rare diplogonadic form of the *Ligula* species (2) not a different genus and not synonymous with the *Ligula* genus, but only another species of the *Ligula* genus. Phylogenetic tree results have displayed differences between clade A and clade B, verifying that the effect of geography in speciation and its effect on molecular structures. *L. intestinalis* and *D. interrupta* samples were different from samples registered in GenBank. Eleven samples of *D. interrupta* and eight samples of *L. intestinalis* were located in a common cluster so that it may be support the analogy of this genus. Moreover, the discrepancy amount of 1% to 4%, shown in the sequence of *L. intestinalis* samples, were compared with each other in the same host that could supplement the presence of a species and/or strains in this genus much like results derived by other researchers (12,13).

## Conclusion

ITS2 may be one of the most useful markers used to distinguish *L. intestinalis* from *D. interrupta.* It can also be used to detect the variations within *L. intestinalis*. Further, *D. interrupta* may be another species of the *Ligula* genus. On the other hand, they could not be different genera because of low levels of nucleotide variation arising from the possibility of polymorphism and therefore *D. interrupta* is another species of the *Ligula* genus. Its possible presence as a species and/or strains in the *Ligula* genus is based on host specificity.
